# Sublethal Effects of Abamectin and Acetamiprid on the Longevity, Fecundity and Detoxification Enzyme Activity of *Rhopalosiphum padi*

**DOI:** 10.3390/insects16060629

**Published:** 2025-06-15

**Authors:** Bokun Wang, Hongming Hui, Xingye Li, Xueqing Yang, Yuting Li

**Affiliations:** 1 College of Plant Protection, Shenyang Agricultural University, Shenyang 110866, China; 2 Key Laboratory of Economical and Applied Entomology of Liaoning Province, Shenyang 110866, China

**Keywords:** *Rhopalosiphum padi*, sublethal effects, abamectin, acetamiprid, enzyme activity

## Abstract

The bird cherry-oat aphid *Rhopalosiphum padi* (L.) poses a significant threat to wheat production, resulting in substantial yield reductions. Abamectin and acetamiprid are frequently utilized for management. This study assessed the sublethal effects of abamectin and acetamiprid on *R. padi* through life table analysis and enzyme activity assays. At 24 h, the LC_10_ and LC_30_ values for abamectin to *R. padi* were 0.063 mg/L and 0.252 mg/L, respectively, while, for acetamiprid, the corresponding values were 0.065 and 0.293 mg/L. The results indicated that exposure to sublethal concentrations of abamectin (AB-LC_10_) extended the longevity of *R. padi* F_0_ generation, while acetamiprid (AC-LC_10_ and AC-LC_30_) decreased it. Furthermore, the fecundity of the F_0_ generation was significantly reduced following exposure to AB-LC_30_, AC-LC_10_ and AC-LC_30_. In the F_1_ generation, exposure to sublethal concentrations of acetamiprid negatively impacted on *R. padi*, as evidenced by a significant reduction in longevity; fecundity and population parameters (*R*_0_, *r*, *λ*, *s_xj_*, *l_x_*, *l_x_m_x_*, *v_xj_* and *e_xj_*). Conversely, sublethal concentrations of abamectin did not significantly affect these parameters. Additionally, population projections revealed a significantly smaller total population size of *R. padi* in the acetamiprid-exposed group compared to both the abamectin-exposed and control groups. Except these population-level effects, the activities of detoxification enzymes, including cytochrome P450 monooxygenases (P450), glutathione S-transferases (GST) and carboxylesterases (CarE), changed differently after treatments. These results suggest that sublethal concentrations of acetamiprid, but not abamectin, significantly inhibit the population growth of *R. padi*. These insights are crucial for *R. padi* control and facilitate the development of effective control strategies that take into account these sublethal effects in integrated pest management strategies targeting *R. padi*.

## Simple Summary

This study investigates the sublethal effects of abamectin and acetamiprid on the growth, development and reproduction of the bird cherry-oat aphid *Rhopalosiphum padi* (L.) (Hemiptera: Aphididae). *R. padi* is an important agricultural pest that poses a significant threat to cereal crops worldwide. We analyzed the life table and enzyme activity to understand the impact of sublethal concentrations (LC_10_ and LC_30_) of abamectin and acetamiprid on *R. padi*. Our findings demonstrate that sublethal concentrations of abamectin extended the longevity of *R. padi* F_0_ generation, whereas acetamiprid significantly reduced both longevity and fecundity. In the F_1_ generation, acetamiprid exposure markedly decreased adult longevity, fecundity and critical population parameters, while abamectin showed no significant effects on these metrics. Population projections further revealed substantially smaller total population sizes in the acetamiprid-exposed groups compared to the abamectin-treated and control groups. Furthermore, detoxification enzyme activities changed differently after treatments. These results indicate that sublethal concentrations of acetamiprid effectively suppress *R. padi* population growth, whereas abamectin exhibits limited inhibitory effects. This highlights the importance of considering sublethal impacts in optimizing integrated pest management strategies against *R. padi*.

## 1. Introduction

The bird cherry-oat aphid, *Rhopalosiphum padi* (L.) (Hemiptera: Aphididae), is a destructive pest of cereal crop worldwide [1,2] and listed as a Category I crop pest by the Ministry of Agriculture and Rural Affairs of China (MARAC) in Announcement No. 333 [3]. In recent years, *R. padi* populations showed a trend of spreading from the southern to northern regions of China, gradually becoming the dominant wheat aphid species [4]. *R. padi* not only causes direct damage by sucking sap but also transmits barley yellow dwarf virus (BYDV), which can result in severe yield loss during outbreaks [5]. Although various management strategies exist, chemical insecticides remain the primary method for controlling wheat aphids in the field [5,6].

Abamectin, a biological pesticide, is widely used to control red spiders and aphids in wheat [7], while acetamiprid, a neonicotinoid insecticide, is extensively applied to control various aphids on crops [8,9]. In field applications, insecticides not only exert lethal effects on target pests but may also decline to sublethal concentrations over time due to environmental degradation and spatial heterogeneity [10]. However, the continuous selection under a long-term low or sublethal dose increases the risk of pests developing resistance to insecticides [10,11]. Indeed, field populations of *R. padi* have developed different levels of resistance to abamectin and acetamiprid, resulting in reduced efficacy [5,8]. Consequently, investigating the sublethal effect of insecticides on insects is crucial for both integrated pest management (IPM) strategies and insecticide resistance management.

Besides insecticide resistance, exposing insects to low or sublethal concentrations of insecticides may impact the behavior and physiological traits of surviving individuals that affect population dynamics [12,13,14]. The sublethal concentration of certain insecticides had negative impacts on the survival rate, developmental period, longevity, fecundity, behavior and demographic parameters [10,14]. For example, thiamethoxam at sublethal doses reduces the feeding behavior of *R. padi* [6,15]. Similarly, exposed to a sublethal concentration of acetamiprid significantly reduced the longevity, fecundity, population life-table parameters and population growth of two wheat aphids, *Sitobion miscanthi* Fabricius and *Schizaphis graminum* (Rondani) [16]. Conversely, some insecticides at sublethal doses may induce hormetic effects by the stimulation of biological processes [17,18]. Such hormetic effects have been documented in various aphids, including *Aphis gossypii* Glover exposed to imidacloprid, acetamiprid and thiamethoxam [19,20,21]; *Myzus persicae* (Sulzer) exposed to imidacloprid and flupyradifurone [22,23]; *Aphis craccivora* (Koch) exposed to flupyradifurone [24] and *S. graminum* exposed to thiamethoxam [25]. Additionally, effects induced by sublethal concentrations and doses of insecticides can be observed in subsequent progeny, even in the absence of direct insecticide exposure in the offspring generation [13,14,26,27]. These findings indicate that the sublethal effects of insecticides are variable and dependent on both the specific insecticide and the target pest. Therefore, understanding these sublethal and transgenerational effects is crucial for optimizing the rational and sustainable use of insecticides, as well as for improving IPM strategies.

In addition, sublethal concentrations of insecticides alter the activity of detoxifying enzymes such as cytochrome P450 (P450), glutathione *S*-transferase (GST) and carboxylesterase (CarE). Alterations in these enzyme activities serve as a biomarker for assessing insecticide exposure [28,29,30]. While the sublethal effects of abamectin and acetamiprid have been extensively studied in various agricultural pests [9,16,26,31], their potential sublethal effects on *R. padi* remain largely undocumented. Therefore, this study aimed to characterize the effects of two sublethal concentrations (LC_10_ and LC_30_) of abamectin and acetamiprid on the development, fecundity and detoxification enzyme activity of *R. padi*. The results not only contribute to the development of scientific and effective pest management strategies but also significantly enhance our understanding of sublethal effects.

## 2. Materials and Methods

### 2.1. Insects

*R. padi* used in the experiment was obtained from a laboratory colony maintained by Prof. Maohua Chen at the College of Plant Protection, Northwest A&F University (Yangling, China). Since 2019, the *R. padi* colony has been continuously reared in the laboratory on wheat seedlings (Changfeng 2112) without exposure to insecticides. The aphids were maintained in a climate chamber (Rxz-380B, Ningbo Jiangnan Instrument Factory, Ningbo, China) under controlled conditions: a temperature of 24 ± 1 °C, relative humidity of 60 ± 5% and a photoperiod of L:D = 16 h:8 h.

### 2.2. Insecticides and Reagents

Abamectin (97% pure) and acetamiprid (98% pure) were purchased from Hebei Weiyuan Biochemical Pesticide Co., Ltd., Shijiazhuang, China. The analytical-grade acetone, ethanol absolute and trisodium phosphate were obtained from Sinopharm Chemical Reagent Co., Ltd., Shanghai, China, and CDNB, Triton X-100, Tris-HCl, reduced glutathione and 7-ethoxycoumarin were sourced from Beijing Solarbio Science & Technology Co., Ltd., Beijing, China. The carboxylesterase (CarE) assay kit was purchased from the Nanjing Jiancheng Bioengineering Institute, Nanjing, China.

### 2.3. Bioassay

To assess the toxicity of abamectin and acetamiprid to *R. padi*, a leaf-dipping method was employed [32]. Stock solutions of abamectin and acetamiprid (10 g/L) were initially prepared by dissolving the insecticides in acetone. Subsequently, these stock solutions were diluted with aqueous solutions containing 0.01% Triton X-100 to create a range of concentrations. Wheat leaves with wingless adult aphids were then completely immersed in the prepared insecticide solutions for 10 s. Following immersion, excess solution was carefully removed using clean filter paper. The treated leaves, along with the aphids, were placed in a square plastic petri dish (10 × 10 cm) containing moistened filter papers to maintain humidity. These petri dishes were then returned to an incubator maintained under the conditions described previously. As a control, the aphids were treated with a solution of 0.01% Triton X-100 only. Each treatment was repeated three times, using at least 30 aphids on 10 leaves (10 cm) from 7–10-day-old wheat seedlings. Mortality data were recorded after 24 h of exposure, and concentration–mortality regressions were estimated by probit analysis using the PROBIT procedure in SAS statistical software version 9.4 (SAS Institute Inc., Cary, NC, USA) [33]. Aphids were considered dead if they failed to response when lightly touched with a fine brush. The LC_10_ and LC_30_ concentrations of abamectin and acetamiprid were chosen for evaluating the sublethal effects on the population parameters of *R. padi*.

### 2.4. Sublethal Effects on the Parent Generation of R. padi

The newly emergence apterous adults of *R. padi* were treated at sublethal concentrations (LC_10_ and LC_30_) of abamectin and acetamiprid, as determined in the toxicity assay, respectively. Aphids treated with 0.01% Triton X-100 only were set as the control. No less than 30 aphids were treated at each concentration, and each treatment was replicated 3 times. Treated aphids were individually transferred to petri dishes containing wheat leaves and incubated under the same environmental conditions. Fresh untreated wheat leaves were replaced daily (24 h) after treatment. The survival and nymphs produced were observed and recorded every 24 h until death.

### 2.5. Sublethal Effects on the Traits of the Offspring of R. padi

To evaluate the transgenerational effects of sublethal concentrations of abamectin and acetamiprid on the offspring, nymphs laid by adults under different treatments were collected. Namely, following the experimental protocol detailed above, adult parent aphids were exposed to sublethal concentrations of abamectin and acetamiprid. Twenty-four hours post-treatment, the surviving adults were individually transferred to new wheat leaves and maintained in separate plastic dishes. After a further 24 h, all adult aphids were removed, leaving only a single neonate nymph on each wheat leaf. Three groups of 30 nymph each were supplied with untreated wheat leaves until death. The aphids were examined every 12 h before molting and the onset of reproduction. Once the aphids reach adulthood, the nymphs produced every 24 h were removed using a fine brush and counted.

### 2.6. Age-Specific Life Table and Population Projection

The raw life table data of individual *R. padi* were analyzed based on the age stage, two-sex life table theory and utilized the method described by Chi (2023) [34]. To estimate the variance and standard error of the biological and population parameters, the bootstrap technique was employed, with 100,000 bootstraps performed [35]. The age stage-specific survival rate (*s_xj_*) represents the probability of *R. padi* surviving to age *x* and stage *j*; age-specific survival rate (*l_x_*) denotes the probability of the *R. padi* population surviving to age *x*; age-specific fecundity of females (*f_xj_*) signifies the mean number of offspring produced by a female *R. padi* at age x; age-specific total oviposition (*m_x_*) represents the mean number of offspring produced by an individual *R. padi* at age *x*. These parameters were calculated as referred from Bai et al. [36]. We used Consume-MSChart and Timing-MSChart to project the growth of different treatments over the next 30 days from an initial population [37].

### 2.7. Determination of Detoxification Enzyme Activities in R. padi

The enzyme activity was determined using the surviving parent adults at 24 h post-exposure. The activity of the P450 enzyme and GST enzyme was determined according to the method described by Hu et al. [38]. CarE enzyme activity was measured using the carboxylesterase activity assay kit (Nanjing Jiancheng Bioengineering Institute, Nanjing, China, Catalog No.: A133-1-1). The protein concentration was determined using the BCA Protein Assay Kit (Takara Biotechnology (Dalian) Co., Ltd., Dalian, China). Each crude enzyme, consisting of a homogenate of 20 apterous adult aphids, was replicated three times.

### 2.8. Data Analysis

The longevity and fecundity of the parent generation were analyzed for significance under different concentrations of abamectin and acetamiprid using one-way analysis of variance (ANOVA) with Tukey’s honest significant difference (HSD) tests. The software SAS version 8.1 was employed for this analysis. The developmental duration of each instar, lifespan, intrinsic rate of increase (*r*), net reproductive rate (*R*_0_), finite rate of increase (*λ*) and mean generation time (T) of the F_1_ generation were compared under different sublethal concentrations of AB and AC using the paired bootstrap test (*p* < 0.05). All data were presented as the mean of three replicates ± the standard error (SE). The survival curves and enzyme activities were plotted with GraphPad Prism software (version 10). The survival rates (*l_x_*), fecundities of female (*f_x_*), fecundities of population (*m_x_*), the net reproductive rate (*l_x_m_x_*), reproductive values (*v_xj_*) and predicted population dynamics of *R. padi* under different treatments were plotted with SigmaPlot version 12.5.

## 3. Results

### 3.1. Determination of the Toxicity of Abamectin and Acetamiprid to R. padi

The toxicity of abamectin and acetamiprid to *R. padi* adults is shown in Table 1. Analysis of the concentration–mortality data reveals that the LC_10_ and LC_30_ values for abamectin to *R. padi* were 0.063 mg/L (AB-LC_10_) and 0.252 mg/L (AB-LC_30_), respectively. In comparison, the LC_10_ and LC_30_ values for acetamiprid were 0.065 (AC-LC_10_) and 0.293 (AC-LC_30_) mg/L, respectively.

### 3.2. Sublethal Effects on the Survial, Longevity and Fecundity of the F_0_ Generation of R. padi

**Figure 1:**
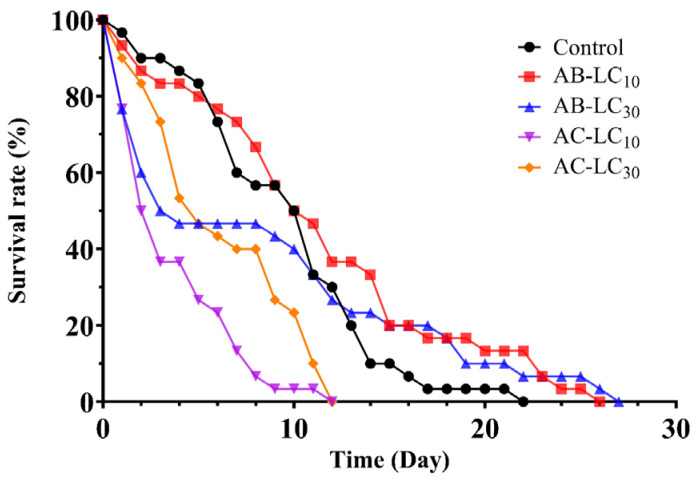
Survival curves of *Rhopalosiphum padi* adults under sublethal concentrations of abamectin and acetamiprid.

The survival curve of adult aphids exposed to sublethal concentrations of abamectin and acetamiprid is illustrated in Figure 1. All curves display a downward trend, in which the survival rate of the AC-LC_10_ and AC-LC_30_ treatment groups declined more rapidly over time than that of the control group. The survival rate of the AB-LC_30_ treatment group dropped sharply within 5 days after treatment and then tended to be gentle and higher than that of the control group. Similarly, the AB-LC_10_ treatment group was consistent with the control group within 12 days post-treatment and thereafter higher than the control group.

For the F_0_ generation of *R. padi*, the longevity and fecundity of the adults were significantly affected by the sublethal concentrations of abamectin and acetamiprid that are evaluated in Table 2. The longevity was significantly extended after exposure to AB-LC_10_, and fecundity was significantly reduced after exposure to AB-LC_30_ compared to the control group. However, the longevity and fecundity were significantly decreased after treatment with AC-LC_10_ and AC-LC_30_ compared to the control group.

### 3.3. Sublethal Effects on the Development and Fecundity of the F_1_ Generation of R. padi

There are different effects of abamectin and acetamiprid treatments on the development and reproduction of the F_0_ generation of *R. padi* in Table 3. Compared to the control, the AB-LC_10_ treatment significantly prolonged the average development period of the nymphal stage in *R. padi* F_1_ generation by 0.33 d, but the development times of the first to fourth instar nymphs were not significant between them. The AB-LC_10_ treatment significantly extended the average development period of the nymphal stage in the *R. padi* F_1_ generation by 0.33 d compared to the control, although there were no significant differences in the development times of the first to fourth instar nymphs. The AB-LC_30_ treatment significantly shortened the development times of the second and third instar nymphs by 0.21 and 0.13 d compared to the control, respectively. Except for the first instar nymphs, the AC-LC_10_ and AC-LC_30_ treatments significantly extended the development period of the second to fourth instar nymphs and nymphal stage in the *R. padi* F_1_ generation compared to the control. The adult longevity, total longevity and oviposition period of the *R. padi* F_1_ generation significantly increased in the AB-LC_10_ and AB-LC_30_ treatments but significantly decreased in the AC-LC_10_ and AC-LC_30_ treatments compared to the control. In addition, fecundity of the *R. padi* F_1_ generation decreased significantly after the AC-LC_10_ and AC-LC_30_ treatments, while there was no significant difference between the AB treatment and control.

### 3.4. Sublethal Effects on the Population Parameters of the R. padi F_1_ Generation

The sublethal effects of abamectin and acetamiprid on the life table of the *R. padi* F_1_ generation are shown in Table 4. Compared to the control, the mean generation time (*T*) was significantly prolonged under all treatments (F = 6.555; *df* = 4; *p* < 0.001). Abamectin treatments (AB-LC_10_ and AB-LC_30_) showed no significant effect on the life table parameters of the *R. padi* F_1_ generation, except the mean generation time (*T*). In contrast, acetamiprid treatments (AC-LC_10_ and AC-LC_30_) significantly negatively affected the life table parameters of the *R. padi* F_1_ generation.

**Figure 2:**
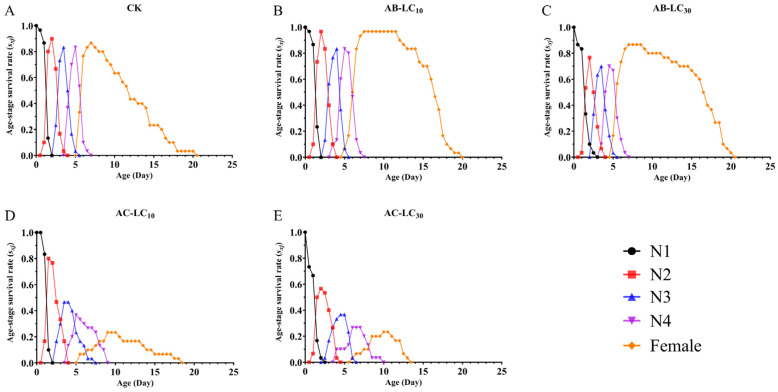
Age stage-specific survival rates (*s_xj_*) of the *Rhopalosiphum padi* F_1_ generation under sublethal concentrations of abamectin and acetamiprid. Abbreviations: CK, untreated control; N1–N4 refers to the 1st to 4th nymphs. (**A**) The *s_xj_* of untreated control group, (**B**) The *s_xj_* of AB-LC_10_ treatment group, (**C**) The *s_xj_* of AB-LC_30_ treatment group, (**D**) The *s_xj_* of AC-LC_10_ treatment group, (**E**): The *s_xj_* of AC-LC_30_ treatment group.

**Figure 3:**
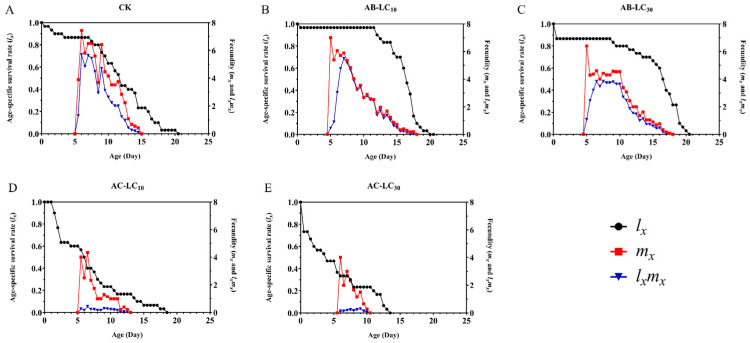
Age-specific survival rate (*l_x_*), age-specific fecundity of the total population (*m_x_*) and age-specific maternity (*l_x_m_x_*) of the *Rhopalosiphum padi* F_1_ generation under sublethal concentrations of abamectin and acetamiprid. (**A**) The *l_x_*, *m_x_* and *l_x_m_x_* of untreated control group, (**B**) The *l_x_*, *m_x_* and *l_x_m_x_* of AB-LC_10_ treatment group, (**C**) The *l_x_*, *m_x_* and *l_x_m_x_* of AB-LC_30_ treatment group, (**D**) The *l_x_*, *m_x_* and *l_x_m_x_* of AC-LC_10_ treatment group, (**E**): The *l_x_*, *m_x_* and *l_x_m_x_* of AC-LC_30_ treatment group. Abbreviations: CK, untreated control.

**Figure 4:**
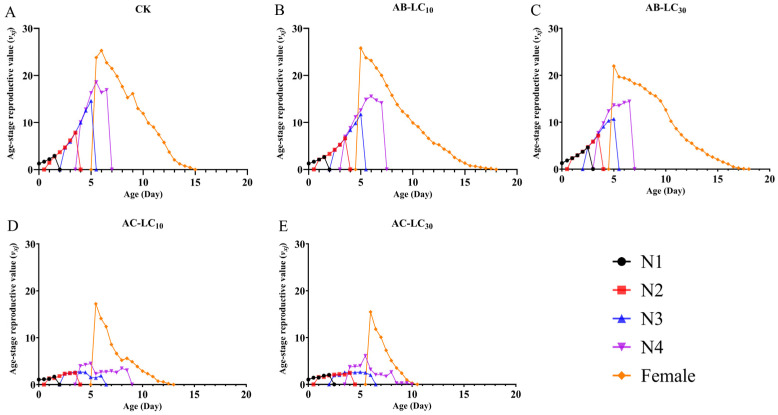
Age stage-specific reproductive rate (*v_xj_*) of the *Rhopalosiphum padi* F_1_ generation under sublethal concentrations of abamectin and acetamiprid. (**A**) The *v_xj_* of untreated control group, (**B**) The *v_xj_* of AB-LC_10_ treatment group, (**C**) The *v_xj_* of AB-LC_30_ treatment group, (**D**) The *v_xj_* of AC-LC_10_ treatment group, (**E**): The *v_xj_* of AC-LC_30_ treatment group. Abbreviations: CK, untreated control; N1–N4 refers to the 1st to 4th nymphs.

The maximum age stage-specific survival rate (*s_xj_*) of *R. padi* exposure to the control, AB-LC_10_, AB-LC_30_, AC-LC_10_ and AC-LC_30_ in the adult stages was 0.86, 0.96, 0.86, 0.67 and 0.67, respectively, indicating that only the acetamiprid-treated *R. padi* F_1_ generation reduced *s_xj_* compared to the control (Figure 2). The age-specific survival rate (*l_x_*) was higher in the abamectin-treated F_1_ generation compared to the control group but lower in the acetamiprid-treated *R. padi* F_1_ generation in Figure 3. The age-specific fecundity (*m_x_*) and the age-specific net maternity (*l_x_m_x_*) were similar in the control and abamectin-treated F_1_ generation. However, the *m_x_* and *l_x_m_x_* of AC-LC_10_ and AC-LC_30_ were significantly lower than those of the control in Figure 3. The age stage-specific life expectancy (*e_xj_*) of the acetamiprid-treated *R. padi* F_1_ generation was lower than that of the control group, while the *e_xj_* value of the abamectin-treated F_1_ generation was similar to that of the control group in different stages in Figure 4.

**Figure 5:**
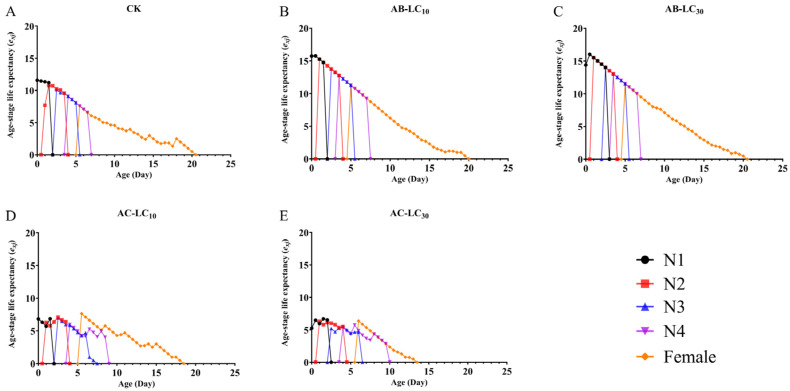
Age stage-specific life expectancy (*e_xj_*) of *Rhopalosiphum padi* F_1_ generation under sublethal concentrations of abamectin and acetamiprid. (**A**) The *e_xj_* of untreated control group, (**B**) The *e_xj_* of AB-LC_10_ treatment group, (**C**) The *e_xj_* of AB-LC_30_ treatment group, (**D**) The *e_xj_* of AC-LC_10_ treatment group, (**E**): The *e_xj_* of AC-LC_30_ treatment group. Abbreviations: CK, untreated control; N1–N4 refers to the 1st to 4th nymphs.

The age stage-specific reproductive values (*v_xj_*) are shown in Figure 5. With the extension of age and stage, *v_xj_* gradually increased at first and then decreased in all the treatments. The peak *v_xj_* values occurred around the age of 5 d in all the treatments. However, the peak *v_xj_* values were 25.29, 25.80, 21.96, 17.23 and 15.48 offspring for the control, AB-LC_10_, AB-LC_30_, AC-LC_10_ and AC-LC_30_, respectively. Moreover, the *s_xj_*, *l_x_*, *m_x_*, *l_x_m_x_*, *e_xj_* and *v_xj_* showed a decrease with the increasing acetamiprid concentration in *R. padi*.

### 3.5. Population Projection of R. padi Exposure to Abamectin and Acetamiprid

**Figure 6:**
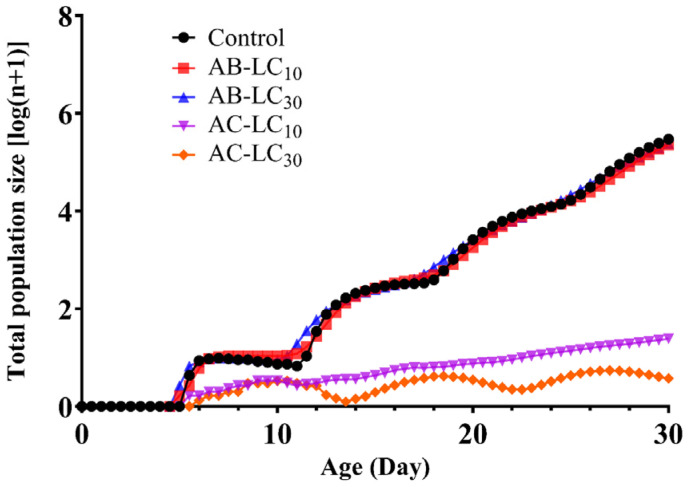
Predicted population dynamics of *Rhopalosiphum padi* over the next 30 days under different treatments.

After beginning with an initial population, the projected population growth of *R. padi* for a 30-d period is shown in Figure 6. The results indicated that *R. padi* could complete four generations under the control treatment and AB-LC_10_ and AB-LC_30_ treatment within the next 30 days. However, only three generations can be completed under the AC-LC_30_ treatment during the same period. The population growth curves in *R. padi* exposure to AC-LC_10_ were nearly linear after 6 days. The population size of *R. padi* exposure to AB-LC_10_ and AB-LC_30_ was similar to the control. However, exposure to AC-LC_10_ (1.7) and AC-LC_30_ (0.9) resulted in a significantly lower population size compared to the control (6.5).

### 3.6. Detoxifying Enzymes Activity of the F_0_ Generation in R. padi

**Figure 7:**
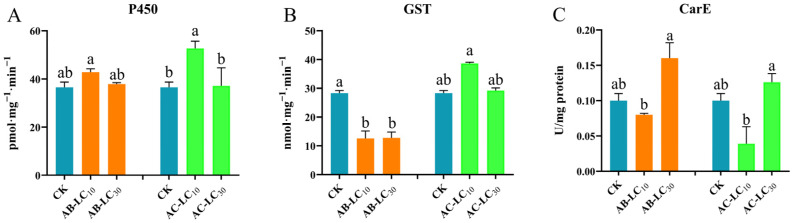
Effects of sublethal concentrations of abamectin and acetamiprid on the detoxification enzyme activities of *Rhopalosiphum padi* from the parent generation. (**A**) P450 enzyme activity, (**B**) GST enzyme activity, (**C**) CarE enzyme activity. Abbreviations: CK, untreated control. The different letters above the bar represent significant differences among these treatments (*p* < 0.05).

The enzyme activity exhibited varied responses following treatment with sublethal concentrations of abamectin and acetamiprid in Figure 7. For abamectin treatments, the activities of P450 showed no significant difference among the treatments and control, while the activities of GST significantly decreased compared to the control. In contrast, CarE activity was significantly higher in the AB-LC_30_ treatment than in the AB-LC_10_ treatment, although it still did not differ significantly from the control. Regarding acetamiprid treatments, the enzyme activities of P450 in AC-LC_10_ showed significant differences compared to AC-LC_30_ and the control, while GST showed no significant differences among the treatments and control. Additionally, CarE activity following acetamiprid treatment showed a similar pattern observed with abamectin.

## 4. Discussion

Arthropod populations are frequently exposed to low and/or sublethal concentrations of pesticides due to the uneven distribution and continuous degradation of active ingredients on plants following the initial pesticide applications [10,39]. These sublethal effects can manifest either as a negative inhibition or positive stimulation of population development and reproduction [10,40]. While many studies have traditionally focused on the effects within a single exposed generation, it is crucial to recognize that sublethal effects can also extend to subsequent generations [21,23,41]. Furthermore, the variability of sublethal effects is influenced by factors such as insect species, insecticide classes and concentrations [42]. In this study, we evaluated the sublethal and transgenerational effects of two insecticides, abamectin and acetamiprid, which are commonly used in wheat fields, on the aphid *R. padi*. Our findings indicate that sublethal concentrations of acetamiprid significantly negatively impacted the survival rate, longevity, development, reproductive and population traits of *R. padi* in both the F_0_ and F_1_ generations. Conversely, sublethal concentrations of abamectin did not exhibit a significant effect on *R. padi*. These results are critical for guiding the rational use of insecticides and for improving resistance management strategies.

Exposure to low or sublethal concentrations of pesticides can affect insect development and reproduction [10]. Our study demonstrated that the adult longevity and fecundity of the F_0_ generation of *R. padi* were significantly diminished when exposed to sublethal concentrations of acetamiprid (Table 2). These results indicate that sublethal concentrations of acetamiprid have adverse effects on *R. padi*, which is consistent with the results of previous research on other neonicotinoid insecticides. For instance, the adult longevity and reproduction of *S. miscanthi* and *S. graminum* were significantly reduced following exposure to a sublethal concentration of acetamiprid [16]. Similarly, the longevity and fecundity of the *R. padi* F_0_ generation were significantly decreased by exposure to sublethal doses (LD_10_, LD_20_ and LD_30_) of dinotefuran [1]. Moreover, studies also found that the development and fecundity of the F_0_ generation of *Brevicoryne brassicae* L. decreased after exposure to sublethal doses of imidacloprid and spirotetramat [13,43]. Many studies have demonstrated the adverse effect of abamectin on the fecundity of insects and mites, such as *Cydia pomonella* (L.) [26], *Grapholita molesta* (Busck) [31], *Neoseiulus longispinosus* (Evans) [44] and *Phytoseiulus persimilis* Athias-Henriot [45]. Similarly, the adult fecundity of the F_0_ generation of *R. padi* was significantly reduced when exposed to LC_30_ concentrations of abamectin. However, the longevity of the F_0_ generation of *R. padi* was significantly prolonged when exposed to the LC_10_ concentration of abamectin, which may be related to hormesis. The longevity extension has also been reported in *Nilaparavata lugens* (Stal) treated with LC_25_ abamectin [46]. Together with reduced fecundity, this suggests that abamectin stress at LC_10_ induces biological tradeoffs, diverting resources from reproduction to survival. In conclusion, the sublethal effects on the parent generation could vary among insecticide classes and concentrations, insect species and application methods [41,47].

Potential transgenerational effects of LC_10_ and LC_30_ of abamectin and acetamiprid on the fitness of *R. padi* were also investigated. Consistent with the results of the F_0_ generation, nymph development, adult longevity and fecundity of the F_1_ generation were significantly reduced following parental exposure to sublethal concentrations of acetamiprid compared to the control. Meanwhile, the population life table parameters, including the *R*_0_, *r*, *λ*, *T*, *s_xj_*, *l_x_*, *l_x_m_x_*, *v_xj_* and *e_xj_*, showed significant decreases in the *R. padi* F_1_ generation-treated parent generation with sublethal concentrations of acetamiprid. These results are consistent with results in other wheat aphid species (*S. miscanthi* and *S. graminum*) after being exposed to sublethal concentrations of acetamiprid [16] and *R. padi* after being exposed to sublethal concentrations of pirimicarb [48]. These results suggest that sublethal concentrations of acetamiprid can inhibit the population of the *R. padi* F_1_ generation. However, low or sublethal doses of insecticides also cause an excitatory effect of toxicants in several pest species, which is beneficial for population development and leads to pest resurgence [11,49]. For instance, the fecundity and *r* of the *Metopolophium dirhodum* (Walker) F_1_ generation were significantly increased under sublethal doses of imidacloprid [50], as well as the *M. persicae* F_1_ generation at sublethal doses of flupyradifurone [23]. The present study observed that the total longevity and *T* of the F_1_ generation tended to be longer in the sublethal concentrations of abamectin treatment groups than in the control. Although no significant changes in fecundity were observed, the prolonged lifespan may lead to an extended period of detrimental effects, potentially resulting in more severe harm. Compared to the control and abamectin, the predicted population dynamics parameters of *R. padi* exposed to sublethal concentrations of acetamiprid exhibited a decline, indicating that low-concentration acetamiprid retains a certain efficacy in controlling *R. padi* populations. The prediction is based on laboratory experimental data for reference, but field conditions, including temperature and natural enemies, may also affect the population dynamics. Therefore, the transgenerational effects of insecticides on pests may influence the population dynamics and outbreaks of pest species and have great significance for IPM programs.

Insects often modulate the activities of detoxification enzymes in response to insecticide-induced stress [32,51]. Enhanced detoxification metabolism is one of the primary mechanisms of insecticides resistance [52]. The activity of detoxifying enzymes may be elevated or descending when insects are exposed to various insecticides. According to Zhang et al. [53], exposure to sublethal doses of metaflumizone and indoxacarb significantly increased the activities of P450 and GST in *Spodoptera frugiperda* (J.E. Smith). Jiang et al. [50] reported that sublethal concentrations of spinetoram treatments inhibited the activities of MFO, GST and CarE in *Tuta absoluta* (Meyrick) larvae. In the present study, exposure to a low concentration of acetamiprid (LC_10_ treatment) led to an increase in P450 enzyme activity in *R. padi*, while the activity of GST was significantly reduced compared to the control. Similarly, sublethal doses of imidacloprid inhibited the activity of the GST enzymes in *S. avenae* and *R. padi* [54]. In contrast, the GST activity was increased in *R. padi* treated by LC_50_ concentrations of chlorpyrifos, isoprocarb, imidacloprid and sulfoxaflor [32]. These seemingly contradictory results suggest that GST activity is influenced by factors such as the specific insecticide used, the exposure dose and the duration of exposure. The observed change in CarE activity may be related to the treated concentration [54]. Therefore, different insecticide types or doses may have varying effects on the activity of detoxification enzymes in insect species.

These findings indicate that sublethal concentrations of acetamiprid effectively restrict the population growth of *R. padi* and remain as a highly effective insecticide for controlling *R. padi* consistent with applications in other wheat aphids: *S. miscanthi* and *S. graminum* [16]. Combined with a slow resistance evolution to acetamiprid in field populations [8], acetamiprid can be a potential management tool for wheat aphid control, particularly in regions where resistance to neonicotinoids and other insecticides has emerged. Although abamectin did not stimulate the fecundity of *R. padi* leading to the resurgence, it still needs to be used with caution in practical applications. Furthermore, to manage *R. padi* effectively and delay resistance, rotate insecticides with different modes of action, avoiding continuous use of the same type for extended periods.

## 5. Conclusions

In this study, we utilized the commonly used concentrations of LC_10_ and LC_30_ to investigate the sublethal impacts of abamectin and acetamiprid on *R. padi*. Abamectin inhibited the reproduction of *R. padi* in the F_0_ generation but had no significant influence on the development and reproduction of the F_1_ generation. Thus, it is important to ensure an appropriate interval and sufficient concentration or dosage when using abamectin. In contrast, acetamiprid is an effective insecticide for suppressing *R. padi* population growth at low concentrations. Sublethal concentrations of acetamiprid significantly reduced the fecundity of both the F_0_ and F_1_ generations, which was confirmed by the reduction in the population size and parameters *R*_0_, *r* and *λ* compared to the control group. Therefore, further field studies are needed to validate these findings and explore the underlying molecular mechanisms. The insights gained from this research may contribute to implement more effective and environmentally friendly pest management programs for *R. padi*.

## Figures and Tables

**Table 1:** Toxicity of abamectin and acetamiprid to adults of *Rhopalosiphum padi*.

Insecticides	n	Slope ± SE	*x* ^2^	*df*	Concentration (mg/L) (95% CI)	
					LC_10_	LC_30_
abamectin	403	1.259 ± 0.158	9.228	3	0.063 (0.034–0.101)	0.252 (0.158–0.348)
acetamiprid	417	1.370 ± 0.177	14.313	3	0.065 (0.023–0.136)	0.293 (0.126–0.457)

CI: Confidence interval.

**Table 2:** Sublethal effects of abamectin and acetamiprid on the longevity and fecundity of the *Rhopalosiphum padi* F_0_ generation.

Parameter	CK	AB-LC_10_	AB-LC_30_	AC-LC_10_	AC-LC_30_
Longevity (d)	9.70 ± 0.88 b	14.63 ± 1.24 a	11.97 ± 1.45 b	4.8 ± 0.54 c	6.3 ± 0.69 c
Fecundity (nymphs/female)	31.37 ± 3.55 a	26.73 ± 3.63 b	20.77 ± 3.77 c	16.90 ± 1.45 d	20.43 ± 1.90 c

Data are shown as the means ± standard error (SE). The different letters within the same row indicate that the treatments were significantly different from each other, as determined using the HSD test (*p* < 0.05). Abbreviations: CK, untreated control.

**Table 3:** Sublethal effects of abamectin and acetamiprid on the developmental period and fecundity of the *Rhopalosiphum padi* F_1_ generation.

Parameters	CK	AB-LC_10_	AB-LC_30_	AC-LC_10_	AC-LC_30_
N1 (d)	1.52 ± 0.05 ab	1.57 ± 0.05 b	1.75 ± 0.08 a	1.46 ± 0.05 b	1.60 ± 0.08 b
N2 (d)	1.44 ± 0.06 b	1.62 ± 0.06 b	1.23 ± 0.06 c	1.89 ± 0.12 a	1.97 ± 0.11 a
N3 (d)	1.42 ± 0.05 b	1.41 ± 0.07 b	1.29 ± 0.06 c	1.88 ± 0.12 a	1.77 ± 0.17 a
N4 (d)	1.48 ± 0.03 b	1.60 ± 0.06 b	1.40 ± 0.05 b	2.31 ± 0.25 a	2.57 ± 0.20 a
Nymph period (d)	5.88 ± 0.07 c	6.21 ± 0.11 b	5.67 ± 0.10 c	7.44 ± 0.51 a	7.93 ± 0.52 a
Oviposition period (d)	5.90 ± 0.44 b	8.47 ± 0.27 a	8.17 ± 0.38 a	2.93 ± 0.76 c	1.92 ± 0.30 c
Adult longevity (d)	13.08 ± 0.68 b	16.26 ± 0.38 a	16.52 ± 0.6 a	6.12 ± 1.32 c	4.43 ± 0.69 c
Total longevity (d)	11.58 ± 0.92 b	15.73 ± 0.64 a	14.38 ± 1.14 a	6.82 ± 0.91 c	5.25 ± 0.85 c
Fecundity (offspring per female)	57.92 ± 3.50 a	55.76 ± 1.24 a	59.73 ± 1.98 a	11.12 ± 3.59 b	6.86 ± 1.91 b

Data are shown as the mean ± standard error (SE). Values in the same row followed by different letters are significantly different when analyzed by the paired bootstrap test (*p* < 0.05). Abbreviations: CK, untreated control; N1–N4 refers to the 1st to 4th nymph.

**Table 4:** Sublethal effects of abamectin and acetamiprid on the demographic parameters of the *Rhopalosiphum padi* F_1_ generation.

Parameters	CK	AB-LC_10_	AB-LC_30_	AC-LC_10_	AC-LC_30_
Net reproductive rate (*R*_0_, nymphs)	50.20 ± 1.92 a	53.90 ± 2.17 a	51.77 ± 2.01 a	2.97 ± 1.27 b	1.60 ± 0.67 b
Mean generation time (*T*, d)	8.15 ± 0.14 b	8.55 ± 0.29 a	8.44 ± 0.17 a	8.70 ± 1.57 a	8.82 ± 2.25 a
Intrinsic rate of increase (*r*, d^−1^)	0.48 ± 0.01 a	0.47 ± 0.01 a	0.47 ± 0.01 a	0.11 ± 0.06 b	0.04 ± 0.06 b
Finite rate of increase (*λ*, d^−1^)	1.62 ± 0.01 a	1.59 ± 0.01 a	1.60 ± 0.01 a	1.12 ± 0.07 b	1.05 ± 0.06 b

Data are shown as the mean ± standard error (SE). Values in the same row followed by different letters are significantly different when analyzed by the paired bootstrap test (*p* < 0.05). Abbreviations: CK, untreated control.

## Data Availability

The original data presented in this study are included in the article. Further inquiries can be directed to the corresponding author.
